# Transcriptional Contribution of Transposable Elements in Relation to Salinity Conditions in Teleosts and Silencing Mechanisms Involved

**DOI:** 10.3390/ijms23095215

**Published:** 2022-05-06

**Authors:** Elisa Carotti, Federica Carducci, Samuele Greco, Marco Gerdol, Daniele Di Marino, Nunzio Perta, Anna La Teana, Adriana Canapa, Marco Barucca, Maria Assunta Biscotti

**Affiliations:** 1 Dipartimento di Scienze della Vita e Dell’Ambiente, Università Politecnica delle Marche, Via Brecce Bianche, 60131 Ancona, Italy; e.carotti@pm.univpm.it (E.C.); f.carducci@univpm.it (F.C.); d.dimarino@univpm.it (D.D.M.); nunzioperta95@gmail.com (N.P.); a.lateana@univpm.it (A.L.T.); a.canapa@univpm.it (A.C.); m.a.biscotti@univpm.it (M.A.B.); 2 Dipartimento di Scienze della Vita, Università degli Studi di Trieste, Via L. Giorgieri, 5, 34127 Trieste, Italy; samuele.greco@phd.units.it (S.G.); mgerdol@units.it (M.G.); 3 New York-Marche Structural Biology Center (NY-MaSBiC), Università Politecnica delle Marche, Via Brecce Bianche, 60131 Ancona, Italy

**Keywords:** transposable elements, fish, silencing mechanisms, salinity, Krüppel-associated box domain zinc finger proteins (KRAB-ZFPs)

## Abstract

Fish are an interesting taxon comprising species adapted to a wide range of environments. In this work, we analyzed the transcriptional contribution of transposable elements (TEs) in the gill transcriptomes of three fish species exposed to different salinity conditions. We considered the giant marbled eel *Anguilla marmorata* and the chum salmon *Oncorhynchus keta*, both diadromous, and the marine medaka *Oryzias melastigma*, an euryhaline organism sensu stricto. Our analyses revealed an interesting activity of TEs in the case of juvenile eels, commonly adapted to salty water, when exposed to brackish and freshwater conditions. Moreover, the expression assessment of genes involved in TE silencing mechanisms (six in heterochromatin formation, fourteen known to be part of the nucleosome remodeling deacetylase (NuRD) complex, and four of the *Argonaute* subfamily) unveiled that they are active. Finally, our results evidenced for the first time a krüppel-associated box (KRAB)-like domain specific to actinopterygians that, together with TRIM33, might allow the functioning of NuRD complex also in fish species. The possible interaction between these two proteins was supported by structural prediction analyses.

## 1. Introduction

A considerable portion of eukaryote genomes is composed of transposable elements (TEs). They are able to move within genomes through transposition using either a DNA intermediate molecule, for example DNA transposons, or an RNA intermediate molecule, known as retrotransposons, that includes Long Terminal Repeats (LTRs) and non LTR elements. These latter comprise Long Interspersed Nuclear Elements (LINEs) and Short Interspersed Nuclear Elements (SINEs).

It is known that TEs can affect genome size, can be co-opted to create novel genes, or be involved in chromosome rearrangements. Not negligible is also the increasing number of evidence reporting a transposition activity that seems to be influenced by abiotic factors such as temperature, salinity, and pH [1,2,3,4,5,6,7,8], suggesting a role of these genetic elements in the regulation of mechanisms responsible for environmental adaptation [2,8,9,10,11]. Moreover, the presence of a specific TE class [10,12] as well as of a particular TE sequence variant [9] has been proposed to be related with species adaptation to a specific environment.

Salinity is an abiotic factor that influences the adaptation of marine and freshwater organisms, and its changes can also represent a possible stress that threatens their survival. Teleosts are an excellent model to investigate the relationship between this environmental parameter and TEs. Indeed, this highly diverse group populates a wide range of habitats across the world, from tropical to polar regions of both sea water (SW) and freshwater (FW) and some species are characterized by the ability to migrate during their lifespan. This fascinating behavior can occur in SW (oceanodromous), in FW (potamodromous) or between these two environments (diadromous). In the latter case, the migratory behavior is carried out for reproductive purposes and organisms have to face, in a defined stage of their life cycle, variations in salinity that require changes in osmotic regulation. Among fish, this aspect is better tolerated by euryhaline species sensu stricto that have acquired the ability to adapt to various salinity conditions.

The giant marbled eel *Anguilla marmorata* is a catadromous species widely distributed in tropical, subtropical, and temperate areas that moves from freshwater to salt water for spawning [13]. The chum salmon *Oncorhynchus keta* is an anadromous species that spends much of its life in salt water and goes up rivers to spawn [14,15]. The marine medaka *Oryzias melastigma* is an euryhaline species able to survive in environments with different salinities.

In this study, we compared the activity of TEs between gill tissues of the considered species analyzing RNA-Seq data available in the public databases obtained from organisms treated at different salinity conditions, FW, brackish water (BW), and SW. Gills are primary organs that are highly sensitive to salinity level, can detect changes in external osmotic pressure, and promote ionic compensatory mechanisms to maintain the osmolarity in body fluids. Moreover, they play crucial roles in physical processes such as gas exchange, nitrogenous waste excretion, and acid-base balance [14,16].

Interestingly, our findings evidenced a variation in TE transcriptional contribution in the case of the giant marbled eel with respect to *O. melastigma* and *O. keta*, and thus the transcriptional activity of genes involved in TE silencing mechanisms was investigated. It is known that to counteract the negative effects due to transposition, host genomes evolved various TE silencing mechanisms involving small RNAs, chromatin and DNA modification pathways, and sequence-specific repressors such as those based on the KRAB-ZFPs recruiting the NuRD complex [11,17]. Here, the evaluation of the expression of genes involved in these mechanisms (six in heterochromatin formation, fourteen known to be part of the NuRD complex, and four of the *Argonaute* subfamily) unveiled that they are active. Intriguingly, our results evidenced for the first time a KRAB-like domain specific of actinopterygians that together with TRIM33 might allow the functioning of NuRD complex.

## 2. Results

### 2.1. Transcriptional Contribution of Transposable Elements in Gill Transcriptomes of A. marmorata, O. melastigma, and O. keta

**Figure 1:**
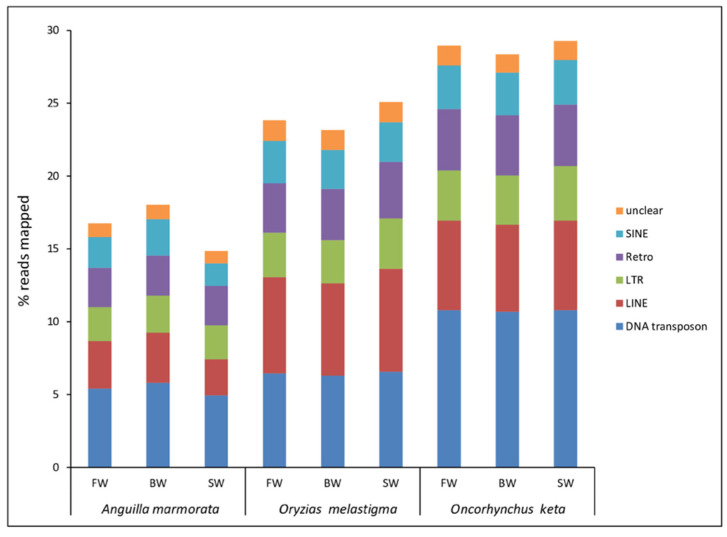
Transcriptional contribution of transposable elements in *Anguilla marmorata, Oryzias melastigma,* and *Oncorhynchus keta* gill transcriptomes. FW: freshwater; BW: brackish water; SW: salt water.

The transcriptional activity of TEs, assessed as percentage of mapped reads, was evaluated through the analysis of gill RNA-Seq data available for *A. marmorata*, *O. melastigma*, and *O. keta* treated in FW, BW, and SW conditions (Figure 1).

The total TE contribution was higher in *O. keta*, followed by that of *O. melastigma* and *A. marmorata*. These differences were mainly ascribable to the activity of LINE retroelements and DNA transposons. In *A. marmorata* and *O. keta*, the highest impact was due to DNA transposons while in *O. melastigma* the two major TE types were DNA transposons and LINE retroelements. The comparison of TE transcriptional contribution showed similar levels between the three tested conditions in *O. keta* (maximum percentage variation of total TEs was 3.22 in the SW vs. BW comparison) and *O. melastigma* (maximum percentage variation of total TEs was 8.31 in the SW vs. BW comparison), while in *A. marmorata* a lower level was detected in the SW condition compared to FW (the increase of percentage variation of total TEs was 12.79) and to BW (the increase of percentage variation of total TEs was 21.43). Regarding percentage variation of single TE class, in *A. marmorata* the main difference was ascribable to SINE retroelements that increased 37.09% in the comparison SW vs. FW and 63.31% in the SW vs. BW comparison. For the other two species considered, minimum percentage variations were reported for the single TE class (Table S1).

### 2.2. Identification and Transcriptional Activity of Genes Involved in TE Silencing Mechanisms

TE silencing mechanisms were investigated in order to explain the different TE contribution observed for the SW condition in the giant marbled eel. The transcriptomes of *A. marmorata*, *O. melastigma*, and *O. keta* were screened to retrieve transcripts with coding sequence (CDS) orthologous to *AGO1, AGO2, AGO3,* and *AGO4* genes of the *Ago* subfamily, to genes encoding proteins involved in heterochromatin formation (*HP1α*, *HP1βa*, *HP1βb*, *HP1γ*, *DNMT1*, *DNMT3Aa*), and in NuRD complex (*CHD3*, *CHD4α*, *CHD4β, HDAC1*, *HDAC2*, *MBD2*, *MBD3b, MTA1*, *MTA2*, *MTA3*, *GATAD2A*, *GATAD2B, RBBP4*, and *RBBP7*) (Table S2).

**Figure 2:**
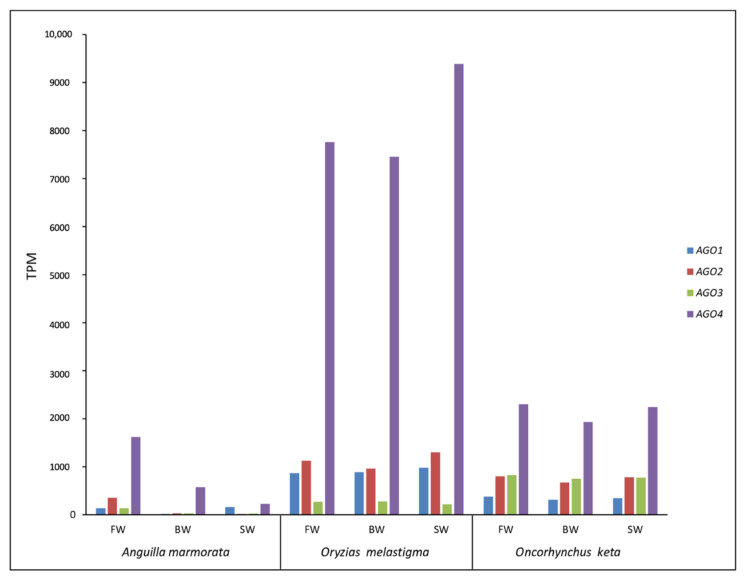
Transcriptional activity of *Argonaute* genes in *Anguilla marmorata, Oryzias melastigma,* and *Oncorhynchus keta* gill transcriptomes. FW: freshwater; BW: brackish water; SW: salt water.

Four transcripts encoding the Ago proteins were retrieved in the analyzed species. *AGO4* of *A. marmorata*, *AGO3* of *O. keta*, and *AGO1*, *AGO2*, and *AGO3* of *O. melastigma* showed a complete CDS. The evaluation of the transcriptional activity of the four *AGO* genes evidenced the same trend between the three tested conditions in chum salmon and in marine medaka, while in giant marbled eel the expression of *AGO* genes was variable between FW, BW, and SW conditions (Figure 2). Overall, *AGO4* showed the highest transcriptional value in all conditions of analyzed species. However, in the case of eel, a decrease in the expression level of this gene was observable from FW to SW condition (Figure 2).

**Figure 3:**
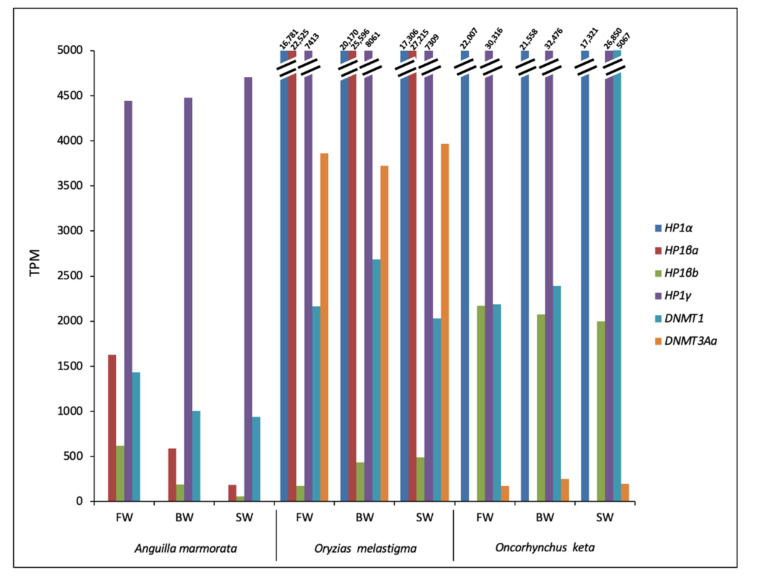
Transcriptional activity of genes involved in heterochromatin formation in *Anguilla marmorata, Oryzias melastigma,* and *Oncorhynchus keta* gill transcriptomes. FW: freshwater; BW: brackish water; SW: salt water.

Concerning genes related to heterochromatin formation, *HP1α* transcript was identified in *O. keta* and *O. melastigma* with a complete CDS; for *HP1β*, two transcripts corresponding to *HP1βa* and *HP1βb* were retrieved in *A. marmorata* and *O. melastigma,* and one transcript corresponding to *HP1βb* was identified in *O. keta*. All *HP1β* sequences presented a complete CDS. For *HP1γ*, a transcript was retrieved in all species (Table S2). This gene was the most expressed in *A. marmorata* and *O. keta*. In the case of *O. melastigma, HP1βa* showed the highest transcriptional levels. The *HP1α* was the second gene having a remarkable transcriptional activity in *O. melastigma* and in *O. keta* (Figure 3). A sequence homologous to *DNMT1* was retrieved in all considered species while *DNMT3Aa* was identified in chum salmon and marine medaka transcriptomes. The expression of *DNMT1* was appreciable in all analyzed RNA-Seq data. Moreover, in *O. melastigma*, among the DNA methyltransferases, the *DNMT3Aa* showed higher transcriptional levels than *DNMT1* (Figure 3).

**Figure 4:**
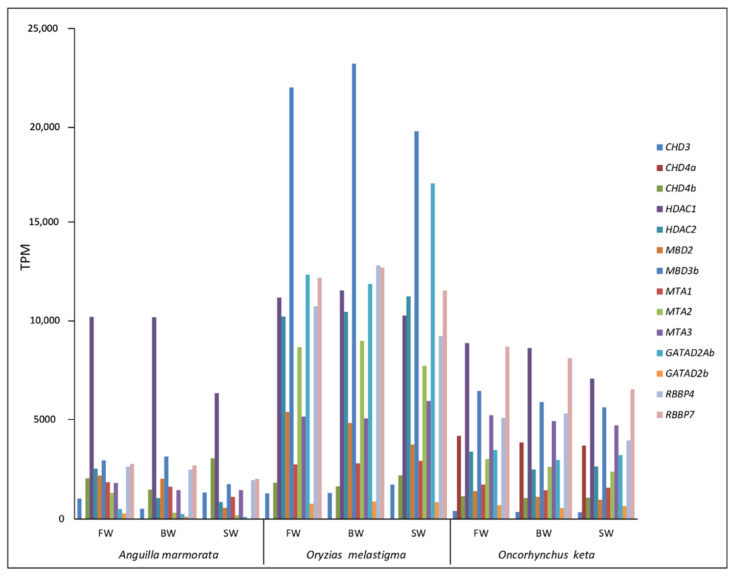
Transcriptional activity of NuRD complex genes in *Anguilla marmorata, Oryzias melastigma*, and *Oncorhynchus keta* gill transcriptomes. FW: freshwater; BW: brackish water; SW: salt water.

Interestingly, for the NuRD complex, the whole set of genes was retrieved in all species investigated. The identified transcripts showed a complete CDS for *HDAC1*, *MBD3b*, *MTA1*, *RBBP4*, and *RBBP7* in *A. marmorata*, for *HDAC2*, *MBD2*, *MBD3b*, *MTA1*, *MTA2*, *RBBP4*, and *RBBP7* in *O. keta*, and for all genes of NuRD complex with the exception of *CHD3* and *CHD4* in *O. melastigma* (Table S2). The expression analysis of the NuRD complex genes showed that they are active in all the three species considered (Figure 4). Moreover, many of them did not show considerable expression variations between the three tested conditions for *O. keta* as well as those for *O. melastigma*. In the case of *A. marmorata*, a different trend was observed between the three conditions with an overall increase in gene expression levels in BW and FW.

**Figure 5:**
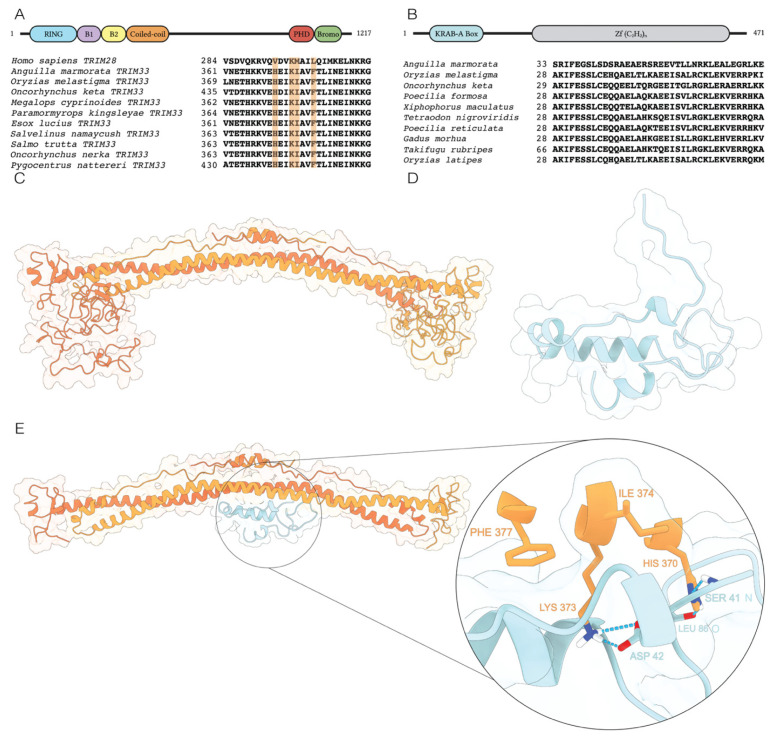
Multiple sequence alignment and 3D structures of the KRAB domain contained in the KRAB-like protein and that of TRIM33 protein. (**A**) In the upper side, a schematic representation of the domain architecture is reported for the *A. marmorata* TRIM33 protein. In the lower side, multiple sequence alignment related to the coiled-coil region of *Homo sapiens* TRIM28 protein and of ten actinopterygian TRIM33 sequences is showed. The main interacting residues at the interface are highlighted in orange. (**B**) In the upper side, a schematic representation of the domain architecture is reported for the *Anguilla marmorata* KRAB-like protein. In the lower side, the N-terminal region multiple sequence alignment of ten actinopterygian KRAB-like sequences is reported. (**C**) Ribbon and surface representation of *A. marmorata* TRIM33 structural model. Chain A and chain B are colored in dark and light orange, respectively. (**D**) Ribbon and surface representation of the putative *A. marmorata* KRAB-like domain. (**E**) Ribbon and surface representation of docked TRIM33/KRAB-like complex with a zoom on the residues H370/K373/I374/F377.

**Figure 6:**
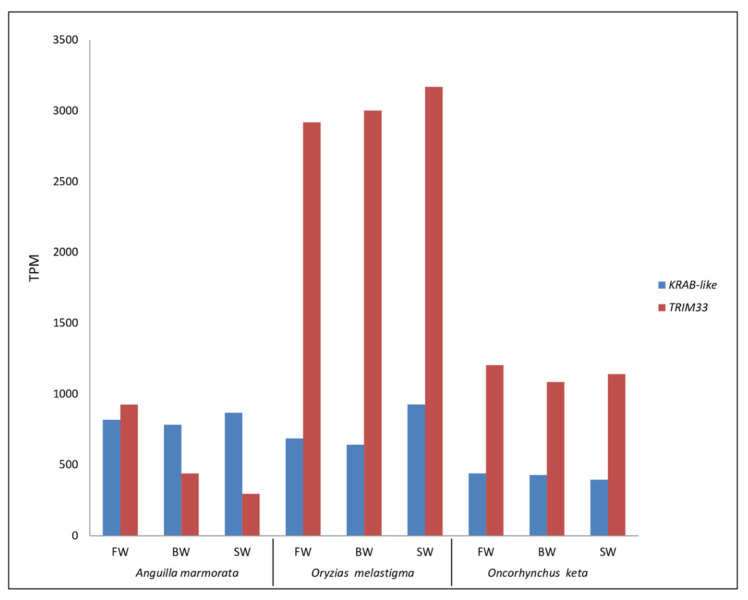
Transcriptional activity of *KRAB*-like and *TRIM33* genes in *Anguilla marmorata, Oryzias melastigma,* and *Oncorhynchus keta* gill transcriptomes. FW: freshwater; BW: brackish water; SW: salt water.

In sarcopterygians, the NuRD complex is recruited at TE sequences through the involvement of KRAB-ZFPs and TRIM28, a protein belonging to the Tripartite Motif family. The former binds the TE sequence through their C-terminal ZF domains and the TRIM28 through their N-terminal KRAB domain, which in turn serves as a scaffold to recruit proteins of the NuRD complex. In actinopterygians, both KRAB-ZFPs and TRIM28 are not present. Therefore, we searched for proteins playing analogous functions in fish species here analyzed. TRIM33 is a protein that has the same domain architecture as TRIM28, being composed of an N-terminal RING Finger/B-Box/coiled coil (RBCC), the Plant Homeo Domain (PHD), and the Bromodomain (BROMO) [18] and is widely spread among ray-finned fish (Figure 5A). The *TRIM33* transcripts showed a complete CDS only for *O. melastigma* (Table S2). Interestingly, our findings highlighted for *TRIM33* the same trend reported for NuRD complex related genes (Figure 6).

Moreover, in the transcriptomes of the considered species, we also searched sequences with a region similar to KRAB domain and ZF motifs. This analysis allowed us to identify a sequence named *KRAB-like* showing these features and widely spread in ray-finned fish (Figure 5B). Its transcriptional expression levels were uniform between the three tested conditions in the two diadromous species, different from *O. melastigma* that showed a higher activity in SW condition (Figure 6).

### 2.3. Structural Prediction of the TRIM33/KRAB-like Complex

To understand the nature of interactions between TRIM33 and KRAB-like proteins we applied a series of structural bioinformatics methodologies. Since there is a lack of structural information available for actinopterygians, TRIM33 was modelled on the human TRIM28 (Figure 5C) while the KRAB-like sequence was modelled on the human KRAB-ZNF93 (Figure 5D). In the wide range of the human ZNFs, we selected ZNF93, since this was identified by Stoll et al. (2019) [19] to strictly bind TRIM28.

We predicted the 3D assembly of the actinopterygian TRIM33/KRAB-like complex (Figure 5E) and used the HADDOCK score to evaluate the poses obtained, it being a valuable parameter of the strength of protein–protein interaction [20,21]. In this case, the HADDOCK score for the best predicted complex was −17.5 +/− 11 with a Z-score of −2.3 for the best prediction. To have a more robust structural reference for the predicted TRIM33/KRAB-like complex, we also built a 3D structure of the *H. sapiens* TRIM28/KRAB-ZNF93 complex.

The superimposition between the human TRIM28/KRAB-ZNF93 and the actinopterygian TRIM33/KRAB-like complexes showed a comparable assembly between both complexes (Figure S1A). Interestingly, the interacting residues of both TRIM28 and TRIM33 were arranged in a similar mode interacting with the cleft of the KRAB domain of KRAB-ZNF93 and KRAB-like, respectively (Figure S1B).

The putative amino acids directly involved in the stabilization of the complex were obtained by combining the results obtained from analyzing the multiple sequence alignment (Figure 5A) between the sequence of human TRIM28 and a set of other actinopterygian TRIM33 sequences and the structural information provided by Stoll et al., (2019) [19]. Indeed, starting from the human TRIM28, we identified the residues H370/K373/I374/F377 for TRIM33. Notably, the H370 and the K373 were oriented into the cleft formed by the KRAB domain helices, where these residues can establish crucial non-covalent interactions (Figure 5E). Moreover, the K373 showed a peculiar role in the TRIM33/KRAB-like complex stabilization since this positively charged residue established an electrostatic interaction (i.e., salt bridge) with D42 (Figure 5E, inset). In order to better characterize the role of K373, we performed a multiple sequence alignment using fifty-one actinopterygian and ten sarcopterygian sequences (Figure S2) to evaluate the degree of conservation of such residue. We found that the K373 was a highly conserved residue both in actinopterygian TRIM33 and sarcopterygian TRIM28.

In addition, we identified other conserved residues from the multiple sequence alignment (highlighted in green in Figure S2), and their positions on the structural model of the complex were shown in Figure S3A,B (i.e., TRIM33 residues: K359/E363/K366/I374/N384/K385/G387/K388; TRIM28 residues: R282/D286/K289/M297/N307/K308/G310/R311).

Altogether these data corroborate our initial hypothesis regarding the ability of TRIM33 and KRAB-like actinopterygian proteins to assemble in a complex comparable to that of humans and thus play a similar biological function.

## 3. Discussion

In this paper, the TE activity was investigated in three fish species showing different salinity tolerance. *A. marmorata*, a catadromous species, and *O. keta*, an anadromous species, migrate for reproduction and thus they have to face changes in salinity in a defined stage of their life cycle. Contrarily, *O. melastigma* is an euryhaline species sensu stricto. Species belonging to *Oryzias* and *Anguilla* genera present a comparable genome size (0.9 pg/N and 1.3 pg/N, respectively) with a TE amount of 40% and 15%, respectively; while species of the *Oncorhynchus* genus show a genome size that is approximately twofold (2.6 pg/N), probably due to the genome duplication event that occurs in salmonids, and TEs occupy a large fraction of their genome with about 40% [10]. Therefore, differences emerging from the comparison of percentage of total TE mapped reads, obtained from the gill transcriptomes of the analyzed species, can be ascribable to their genome size and TE amount. Analyzing the three tested conditions in each species, the lack of remarkable variations in TE contribution in *O. melastigma* might be related to its strong ability to survive in aquatic environments with a wide range of salinity. *O. keta* specimens selected for this study were at the fry stage and thus adapted to freshwater. Therefore, it was interesting to investigate if the exposure at higher salinity concentrations caused changes in TE transcription. However, our findings showed no appreciable differences in TE contribution between the three tested conditions in this species. The lack of this evidence might be attributable to short periods of exposure [14], not sufficient to provoke a TE transcriptional response. An appreciable difference in percentage of TE mapped reads emerged comparing the SW with FW and BW conditions of *A. marmorata*. Since specimens used for this experiment were at the juvenile stage and thus adapted to living in seawater [16], the exposure to low salinity levels might have determined the increase in TE transcription observed. Moreover, the major impact in these changes was due to the expression of SINE retroelements (37.09% for SW vs. FW, 63.31% for SW vs. BW). This finding could be in line also with our previous results that highlighted a role of these kinds of elements in the catadromous behavior of eels [10]. Indeed, it has been reported that SINE retroelements affect gene expression since they are located at the boundaries of transcriptionally active or inactive domains favoring intra- or inter-chromosomal interactions [22,23,24].

The transcriptional activation of TEs is strictly related to silencing mechanisms [11,25,26,27]. Therefore, the activity of genes encoding proteins involved in TE repression was investigated. It is noteworthy that no appreciable differences were detected in the expression levels of genes belonging to the *Ago* subfamily between the analyzed conditions in chum salmon and marine medaka. In giant marbled eel, the expression of these genes was variable and, in particular, that of *AGO4* showed a decreasing level from FW to SW. This gene and *AGO2* showed an expression trend similar to that of TEs. Interestingly, *AGO4* has been shown to play a role in transposon silencing in gonads [28] and our results suggested that this function might also be conserved in somatic tissues. Moreover, a slicing activity for *AGO2* has been reported and the endosiRNAs derived seem to be involved in the transposon repression [29].

Among heterochromatinization related genes, *HP1βb* and *HP1γ* were transcriptionally active in all species and tested conditions. The high expression levels of *HP1γ* in *O. keta* might explain the low difference detected in total TE contribution levels, compared to the other two species. Indeed, a higher TE activity was expected in chum salmon due to the high number of mobile elements present in its genome. Between the two genes selected for *DNMTs*, the expression analysis suggested that DNMT1 might be the candidate protein in TE methylation.

The expression analysis of genes encoding proteins of NuRD complex showed that this system is active in the fish species investigated. To date, to our knowledge, the activity of these genes in fish has been reported in the blastema in fin regeneration [30]. The involvement of NuRD complex in TE silencing in tetrapods has been investigated at the embryonic stage [31,32,33,34]. However, our data obtained from gills of juveniles and adults were in agreement with recent works showing the expression of NuRD complex genes also in adult tissues [35,36]. Overall, the expression of genes investigated did not show remarkable variations between the three tested conditions both in *O. melastigma* and *O. keta* while a major variability was detected in *A. marmorata*. This trend reflected that obtained for TE transcription, suggesting a possible relationship between NuRD complex and TEs also in fish. In tetrapods, the NuRD complex is recruited at TE sequence through the involvement of KRAB-ZFPs and TRIM28. In fish lineage, these components have not been identified. For this reason, we have investigated the possibility that TRIM33 and our KRAB-like could play the same function of TRIM28 and KRAB-ZFPs of tetrapods. Indeed, TRIM33 is a protein belonging to the Tripartite Motif family, it shows the same domain architecture of TRIM28 and is spread among ray-finned fish [18]. Recently, Helleboid and colleagues (2019) [37], analyzing the interactome of KRAB-ZFPs in human, have evidenced that ZFPs might interact with other members of the TRIM family. Intriguingly, our analysis revealed an increased transcription of *TRIM33* with salinity decrease in giant marbled eel, following the same trend observed for genes involved in NuRD complex. This finding supported our hypothesis that in fish TRIM33 might play a role similar to that of tetrapod TRIM28. Moreover, the KRAB-like sequence here reported showed a KRAB-like domain at the N terminus and several Zinc Finger motifs at the C terminus. This protein domain architecture is similar to that of tetrapod KRAB-ZFPs. The docking analysis supported the ability of TRIM33 and KRAB-like proteins to assemble in a complex in actinopterygians, comparable to that of human TRIM28/KRAB-ZFP93.

## 4. Materials and Methods

RNA-Seq raw data were obtained from the public database NCBI GenBank (https://www.ncbi.nlm.nih.gov/ accessed on 21 September 2021) deposited under the accession number GSE95803 for *A. marmorata* [16], in Sequence Read Archive (SRA) under the accession numbers SRX3932910-SRX3932912 for *O. keta* [14], and under the BioProject ID PRJNA745044 for *O. melastigma* [38]. Data were obtained from juvenile specimens in the case of *A. marmorata* and *O. keta* and from adult specimens in the case of *O. melastigma.* Exposures were conducted at FW, BW, and SW (Table S3). For each condition, available data were referred to pooled biological replicates [14,16,38]. Raw paired-end reads were imported in the CLC Genomics Workbench v.12 (Qiagen, Hilden, Germany) and trimmed with the proper internal tool by removing sequencing adapters, low quality bases, and low quality read ends using default parameters. Trimmed reads were then de novo assembled with the proper tool within the CLC using default parameters. Completeness of the de novo assembled transcriptomes was evaluated through BUSCO v5 using the Actinopterygii OrthoDB v10 database as reference [39].

### 4.1. Estimation of TE Activity

To estimate the TE transcriptional activity, we first identified TEs in the de novo assembled transcriptomes with RepeatMasker v.4.1.0 using a custom library created following the methodology described in our previous work [10]. After RepeatMasker analysis, the trimmed reads related to FW, BW, and SW conditions for *A. marmorata*, *O. keta,* and *O. melastigma* were mapped against reference transcriptomes to calculate expression values, using the proprietary *RNA-Seq Analysis tool* included in the CLC Genomics Workbench v.12 to set the following mapping parameters: length fraction = 0.75 and similarity fraction = 0.98. In order to remove redundancy, the RepeatMasker output file was filtered removing entries not classified as TEs, keeping those with the highest score and length values. The overall expression of each TE class was calculated by summing the expression values of each TE type (SINE, Retro, LTR, LINE, DNA transposons, and unclear). The expression values were then transformed in percentage of mapped reads to achieve comparability between species.

### 4.2. Identification and Expression Analysis of Genes of Interest

Focusing on genes of interest, they were retrieved through TBLASTN [40] from the de novo assembled transcriptomes obtained from gills of *A. marmorata*, *O. melastigma*, and *O. keta*. In particular, the search was made for genes involved in heterochromatinization (*HP1α*, *HP1βa*, *HP1βb*, *HP1γ*, *DNMT1*, *DNMT3aa*), genes related to the NuRD complex (*KRAB-like*, *TRIM33*, *CHD3*, *CHD4a*, *CHD4b*, *HDAC1*, *HDAC2, MBD2*, *MBD3b*, *MTA1*, *MTA2*, *MTA3*, *GATAD2ab*, *GATAD2b*, *RBBP4*, *RBBP7*), and four genes of the *Argonaute* subfamily (*AGO1*, *AGO2*, *AGO3*, and *AGO4*). Transcripts were translated using the EMBOSS Transeq translation tool (https://www.ebi.ac.uk/Tools/st/emboss_transeq/ accessed on 18 November 2021) and UTR and CDS regions were identified (Table S2). The aforementioned sequences were deposited in GenBank under the accession numbers listed in the Table S2. To ensure the comparison between species, gene expression values were computed using a scaling factor, based on the cumulative expression of a dataset composed of 3640 orthologs derived from the Actinopterygii OrthoDB v10 database.

In detail, the dataset was created as follows: for each transcriptome of the three species considered in this study, expression levels of genes attributed from BUSCO analyses as “*complete and single copy*” and “*fragmented*” genes were kept as the number of mapped reads; expression values of genes classified as “*duplicated*” (most probably derived from transcriptional isoforms) were computed as the sum of each copy of single BUSCO and the expression levels of “missing” genes were set to 0. This dataset was then used as a calibration set, computing a scaling factor that was applied to the original expression values of the genes of interest as described in Biscotti et al., (2016) [41].

### 4.3. Molecular Modelling

The computational analysis was performed to investigate the putative interaction between TRIM33 and KRAB-like sequence identified in actinopterygians. The three-dimensional (3D) model for TRIM33 was built using SwissModel web server (https://swissmodel.expasy.org/ accessed on 12 January 2022) [42,43,44], while the putative KRAB domain contained in the KRAB-like sequence was built through Modeller web server (https://salilab.org/modeller/, https://swissmodel.expasy.org/ accessed on 12 January 2022) [45]. Because the template used for the modelling of the KRAB domain was a predicted structure of the *Homo sapiens* ZNF93 (UniProtKB accession number AF-P35789), we validated this model comparing it to the NMR murine structure (PDB ID 1V65).

The docking was performed using the HADDOCK web server [46,47]. We selected as active residues (AIRs) the whole TRIM33/TRIM28 coiled-coil region and all the KRAB-like/KRAB-ZNF93 residues pointing out that our docking protocol was completely unbiased. The HADDOCK score was used to classify the poses of the complexes sampled for each docking run. In principle, the lowest HADDOCK score corresponds to the highest predicted protein–protein affinity [47,48].

For the multiple sequence alignment, we used COBALT, a constraint-based alignment tool for multiple protein sequences included in the NCBI toolkit [49].

Molecular graphics and analyses were obtained with UCSF ChimeraX [50].

## 5. Conclusions

TEs represent one of the most intriguing genome components and the analysis of their activity in somatic tissues contributes to understanding the role of these genetic elements in regulating the physiology of organisms [51]. Our data evidenced a variation in TE contribution in the case of juvenile eels, commonly adapted to salty water, when exposed to brackish and freshwater conditions. Indeed, it seems that gill cells activate TE controlling systems in response to the TE increase occurred from salt water to freshwater. Interestingly, our analyses suggested for the first time that besides *Argonaute 4*, *HP*, *DNMT*, the NuRD complex might also be involved in fish TE silencing. Therefore, we propose for the first time the existence of a KRAB-like domain specific to actinopterygians that together with TRIM33 allows the functioning of the NuRD complex also in fish lineage.

## Data Availability

Sequences were deposited in GenBank under the accession numbers provided in the Table S2.

## Supplementary Materials

The following supporting information can be downloaded at: https://www.mdpi.com/article/10.3390/ijms23095215/s1.
