# Supplementary figures and images for: Transcriptional Contribution of Transposable Elements in Relation to Salinity Conditions in Teleosts and Silencing Mechanisms Involved
Source: Int J Mol Sci. 2022 May 6;23(9):5215. doi: 10.3390/ijms23095215 (PMC9101882; doi:10.3390/ijms23095215)

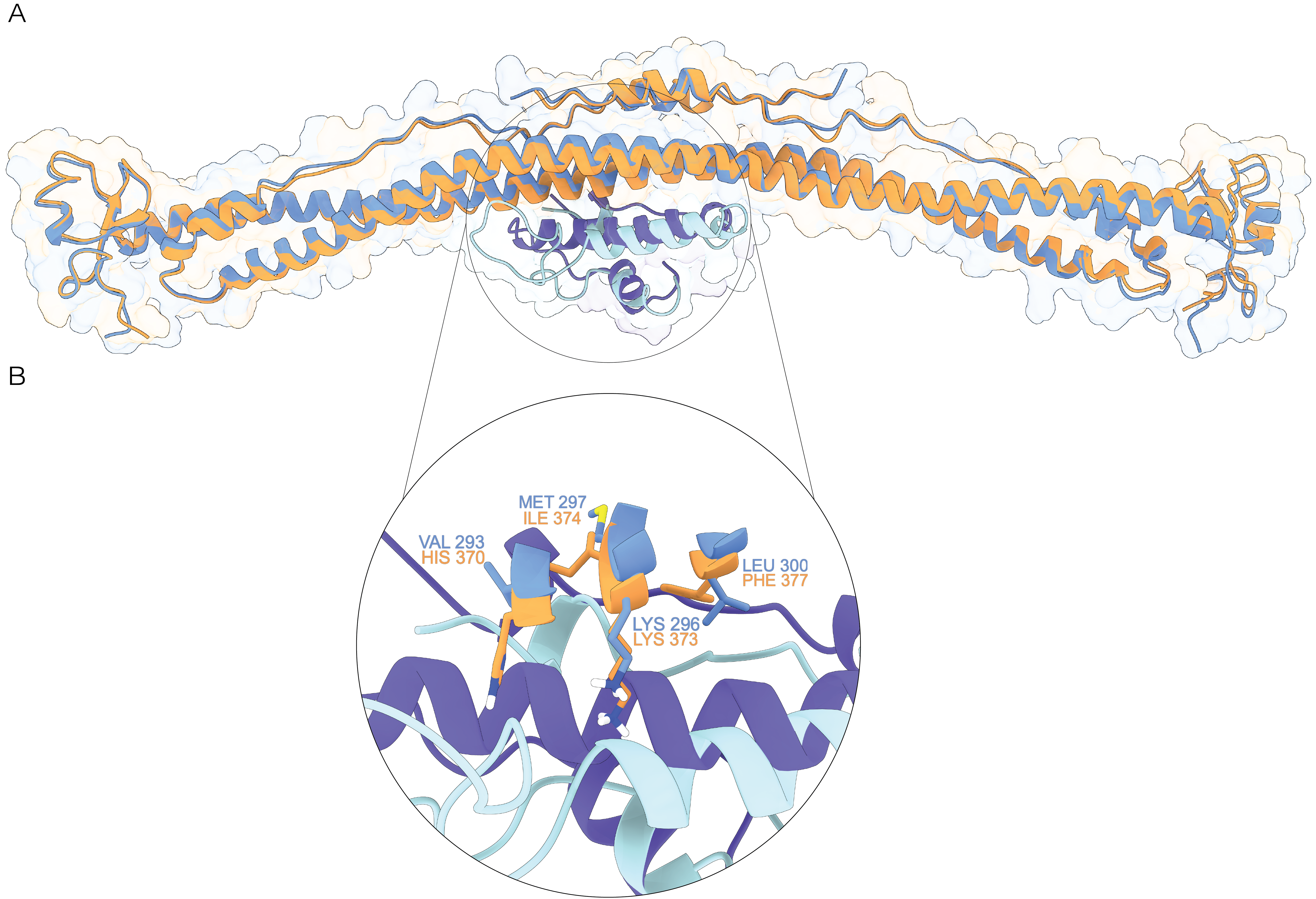

Supplement: Supplementary file 1 [file ijms-23-05215-s001.zip › Supplementary Materials/SuppFigures/FigureS1.png]

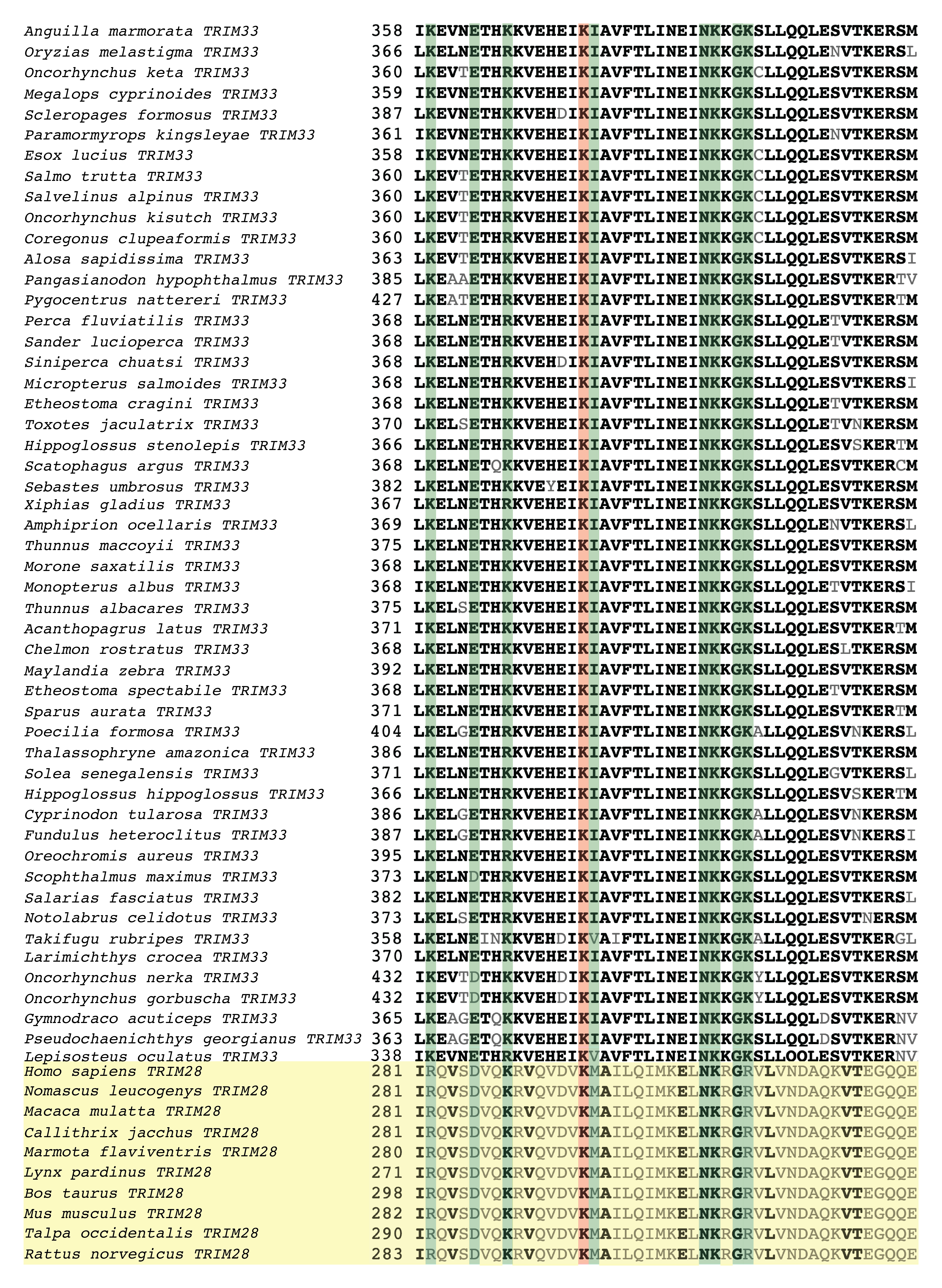

Supplement: Supplementary file 1 [file ijms-23-05215-s001.zip › Supplementary Materials/SuppFigures/FigureS2.png]

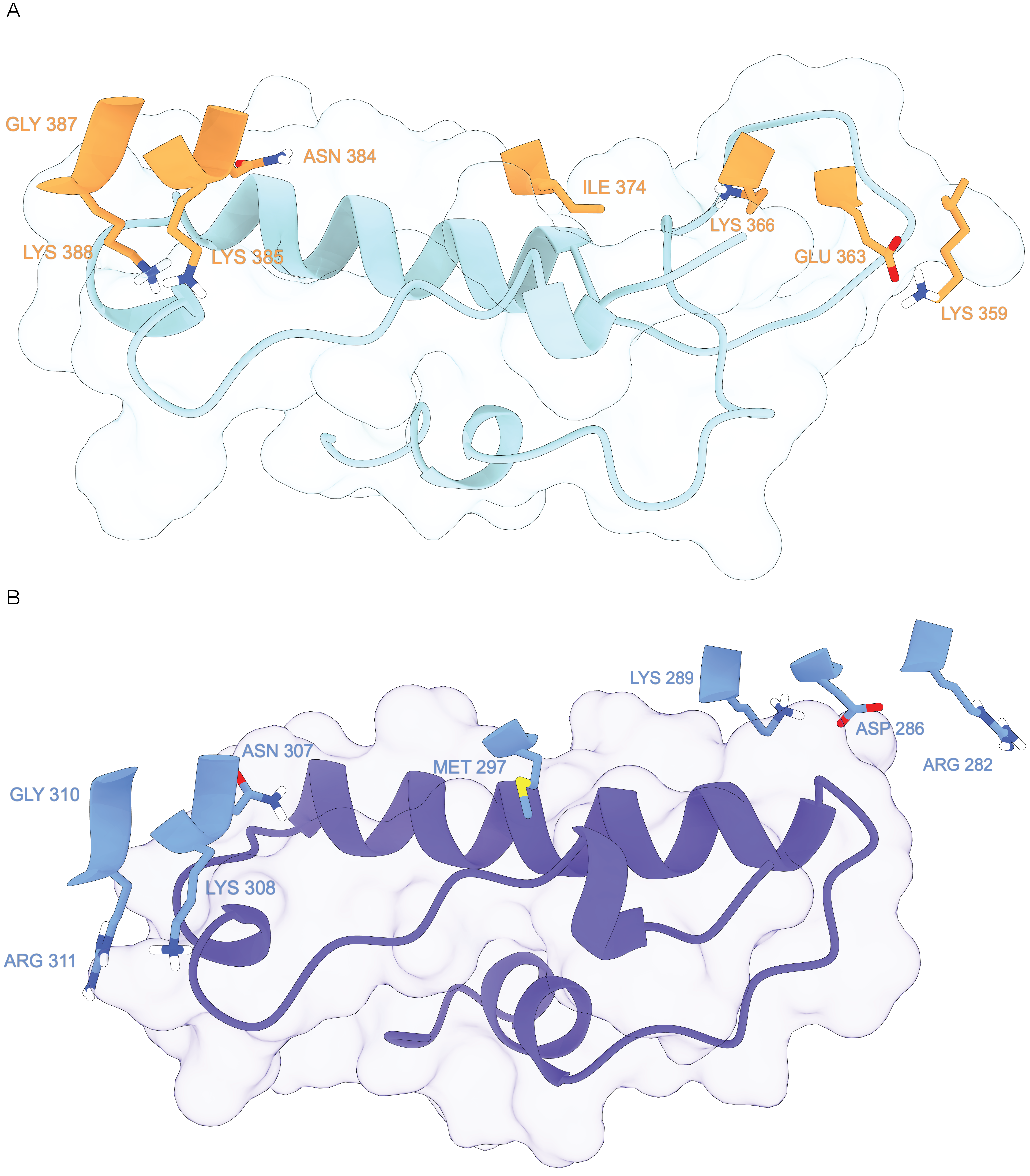

Supplement: Supplementary file 1 [file ijms-23-05215-s001.zip › Supplementary Materials/SuppFigures/FigureS3.png]
